# Development of Gastroretentive Carriers for Curcumin-Loaded Solid Dispersion Based on Expandable Starch/Chitosan Films

**DOI:** 10.3390/molecules28010361

**Published:** 2023-01-01

**Authors:** Worrawee Siripruekpong, Ousanee Issarachot, Kanidta Kaewkroek, Ruedeekorn Wiwattanapatapee

**Affiliations:** 1 Department of Pharmaceutical Technology, Faculty of Pharmaceutical Sciences, Prince of Songkla University, Hatyai 90112, Songkhla, Thailand; 2 Phytomedicine and Pharmaceutical Biotechnology Excellence Center, Faculty of Pharmaceutical Sciences, Prince of Songkla University, Hatyai 90112, Songkhla, Thailand; 3 Pharmacy Technician Department, Sirindhron College of Public Health of Suphanburi, Mueang Suphan Buri District 72000, Suphan Buri, Thailand; 4 Faculty of Integrative Medicine, Rajamangala University of Technology Thanyaburi, Thanyaburi, Khlong Luang 12130, Pathum Thani, Thailand

**Keywords:** curcumin, expandable film, solid dispersion, starch, gastroretentive drug delivery system

## Abstract

Curcumin, a polyphenolic extract from the rhizomes of turmeric, exhibits antioxidant, anti-inflammatory, and anticancer activities, which are beneficial for the treatment of gastric diseases. However, curcumin’s therapeutic usefulness is restricted by its low aqueous solubility and short gastric residence time. In this study, curcumin-loaded solid dispersion (ratio 1:5) was prepared using Eudragit^®^ EPO (Cur EPO-SD), resulting in an approximately 12,000-fold increase in solubility to 6.38 mg/mL. Expandable films incorporating Cur EPO-SD were subsequently prepared by solvent casting using different types of starch (banana, corn, pregelatinized, and mung bean starch) in combination with chitosan. Films produced from banana, corn, pregelatinized and mung bean starch unfolded and expanded upon exposure to simulated gastric medium, resulting in sustained release of 80% of the curcumin content within 8 h, whereas films based on pregelatinized starch showed immediate release characteristics. Curcumin-loaded expandable films based on different types of starch exhibited similar cytotoxic effects toward AGS cells and more activity than unformulated curcumin. Furthermore, the films resulted in increased anti-inflammatory activity against RAW 264.7 macrophage cells compared with the NSAID, indomethacin. These findings demonstrate the potential of expandable curcumin-loaded films as gastroretentive dosage forms for the treatment of gastric diseases and to improve oral bioavailability.

## 1. Introduction

Curcumin is a polyphenolic compound extracted from the rhizomes of turmeric (*Curcuma longa* Linn.) belonging to the Zingiberaceae family. Curcumin has been proven to display numerous pharmacological activities, including anti-inflammatory [1], antioxidant [2] anticancer [3], antiulcer [4], anti-Helicobacter pylori [5] and wound-healing [6] activities. Several studies have shown that curcumin is able to decrease gastric acid secretion and enhance the mucosal defensive mechanism by suppressing iNOS-mediated inflammation [7]. Although, curcumin is stable in the acidic pH environment of the stomach, its utility for treatment of gastric ulcers is limited due to extremely low aqueous solubility and low oral bioavailability resulting from incomplete absorption in the GI tract [8].

The poor aqueous solubility and dissolution rate of curcumin may be improved by the formulation of solid dispersions. This strategy involves intimate mixing of hydrophilic polymers and bioactive compounds, for example, from plants, including curcumin, resveratrol, quercetin, and glycosides [9,10,11,12,13]. The bioavailability of curcumin may be further enhanced by employing gastroretentive drug delivery systems, which are designed to extend residence times in the stomach, resulting in prolonged drug release and thereby improving bioavailability. Among these devices, expandable films have been designed to increase their volume or change shape by swelling or unfolding when in contact with gastric fluids, which prevents premature emptying from the stomach through the pyloric sphincter [14,15].

Biopolymers offer advantages for drug delivery due to their range of physicochemical properties, biodegradability, biocompatibility, and safety. Starch is one of the most widely used natural polymers in medicine formulations [16]. Corn starch in its native and modified forms (pregelatinized starch) has been used extensively as a pharmaceutical excipient, particularly in solid dosage forms, serving as a binder, disintegrant, diluent, and lubricant [17,18]. Banana starch (*Musa sapientum*, belonging to the family Musaceae) has been employed as an excipient for thickening and gelling purposes and also for therapeutic applications [19,20] as an antioxidant, antiulcerogenic, anti-*Helicobacter pylori* and wound-healing agent [21]. Banana starch has been used in Thai traditional medicine to remedy gastrointestinal diseases, and animal studies have confirmed its efficacy in protecting against aspirin-induced gastric ulcers and gastric inflammation [22]. Mung bean starch is produced from legumes of mung bean (*Vigna radiate*, belonging to the Fabaceae family). It contains a high amount of amylose (40%) and exhibits excellent gelling capacity and cohesiveness [23,24].

The utility of starch-based films for gastroretentive drug delivery is limited by poor mechanism properties and water resistance. However, polymer blending with chitosan has been shown to be an effective strategy for the production of novel materials for a variety of applications, and several studies have investigated film formation from blends of chitosan and starch. Chitosan is a natural polysaccharide derivative of chitin obtained by deacetylation (chemical structure: β (1–4) glycosidic linkage of N-acetylglucosamine). It is highly biocompatible and used extensively in the pharmaceutical industry as a film-forming agent, coating, and sustained-release polymer [25,26].

To date, there has been very little research reported on the application of starch blend films as a gastroretentive drug carrier [11,12]. The aim of this study, in particular, was to develop expandable films based on blends of chitosan with mung bean starch, pregelatinized starch, corn starch, or banana starch as a carrier for curcumin solid dispersion prepared using Eudragit^®^ EPO as the hydrophilic polymer. Eudragit^®^ EPO was selected as a suitable carrier for specific delivery to the stomach due to its fast rate of dissolution in the gastric fluid up to a pH of 5.0 [9,10]. The physicochemical characteristics of the curcumin-loaded films were investigated in detail, together with their anti-inflammatory and anticancer activities in cell culture.

## 2. Results and Discussion

### 2.1. Solubility of Curcumin Solid Dispersions

Curcumin exhibits extremely poor aqueous solubility (2.67 µg/mL in distilled water) [27] and high lipophilicity (log *p* value~3.29) [8]. The curcumin solid dispersions were orange–red powders, whereas the curcumin physical mixtures were orange–yellow powders with a particle size range of 50–250 µm.

The solubility in 0.1 N hydrochloric acid (pH = 1.2) of curcumin: Eudragit^®^ EPO solid dispersions (Cur EPO-SD) and physical mixtures (Cur EPO-PM) at ratios of 1:3, 1:5, 1:6, and 1:8 *w*/*w* are shown in Table 1. The solubility of Cur EPO-SD in 0.1 N hydrochloric acid (pH = 1.2) was significantly higher than that of Cur EPO-PM and curcumin. The highest solubility of Cur EPO-SD (6.4 mg/mL) was achieved at a 1:5 *w/w* ratio and was around 12,000-fold higher than that of curcumin (0.52 μg/mL).

The major solubility enhancement was attributed to the intermolecular hydrogen bonding between the hydroxyl group of curcumin and the cationic functional group of Eudragit^®^ EPO, resulting in the formation of soluble complexes [28]. The results support previous reports of curcumin–Eudragit^®^ E100 SD [29,30,31] and Cur EPO-SD [9]. The solubility of curcumin when formulated with Eudragit^®^ EPO at higher *w/w* ratios of 1:6 and 1:8 was reduced, possibly due to steric hindrance of interactions between curcumin and the polymer. In addition, the macromolecule conformation change at high polymer concentration is expected to limit access to curcumin in polymeric cavities, leading to reduced solubility [9].

These findings are in agreement with the solubility of curcumin, glycoside-rich Centella asiatica extract [32], tacrolimus [33], and nimesulide [34] when formulated as solid dispersions with Eudragit^®^ EPO.

### 2.2. Dissolution of Curcumin Solid Dispersions

**Figure 1:**
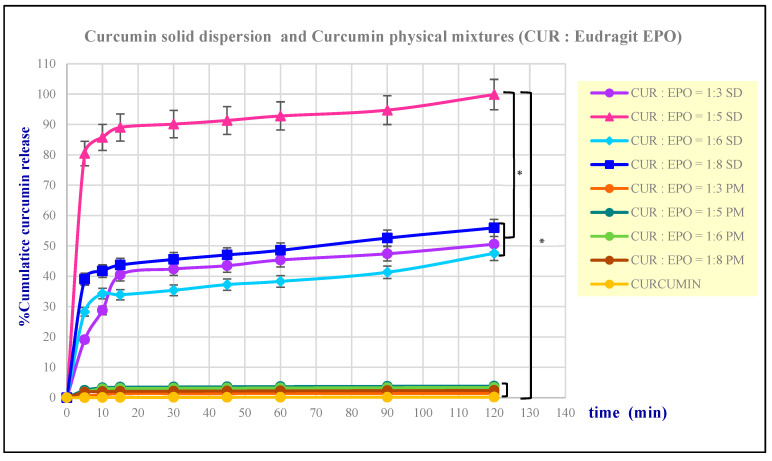
In vitro dissolution studies of curcumin, curcumin–Eudragit EPO solid dispersions and curcumin–Eudragit EPO physical mixtures at various ratios. Note: * denotes *p*-value < 0.01.

The dissolution behavior of Cur EPO-SDs and Cur EPO-PMs in simulated gastric fluid is shown in Figure 1. Physical mixtures resulted in only 1.5–4% dissolution in 2 h, which is similar to curcumin (less than 1%). However, solid dispersions of 1:3, 1:6, and 1:8 *w/w* ratios gave rise to rapid dissolution of approximately 30–45% of the curcumin content in 10–15 min, followed by a gradual dissolution phase, resulting in approximately 50% dissolution at 2 h. In comparison, the solid dispersion at a ratio of 1:5 showed significantly higher curcumin dissolution (around 90%) after 10 min and complete dissolution by 2 h. Cur EPO-SD at a ratio 1:5 was therefore selected for further studies as a result of achieving the highest curcumin dissolution.

These results are in accordance with solubility measurements. The improvement in dissolution of curcumin solid dispersions may be explained by various factors, including particle size reduction, reduced particle aggregation, and the wetting effect of Eudragit^®^ EPO. Moreover, in powder XRD studies, curcumin was shown to be converted to an amorphous state in solid dispersion, as mentioned below.

### 2.3. Fourier Transform Infrared Spectroscopy of Curcumin Solid Dispersions

**Figure 2:**
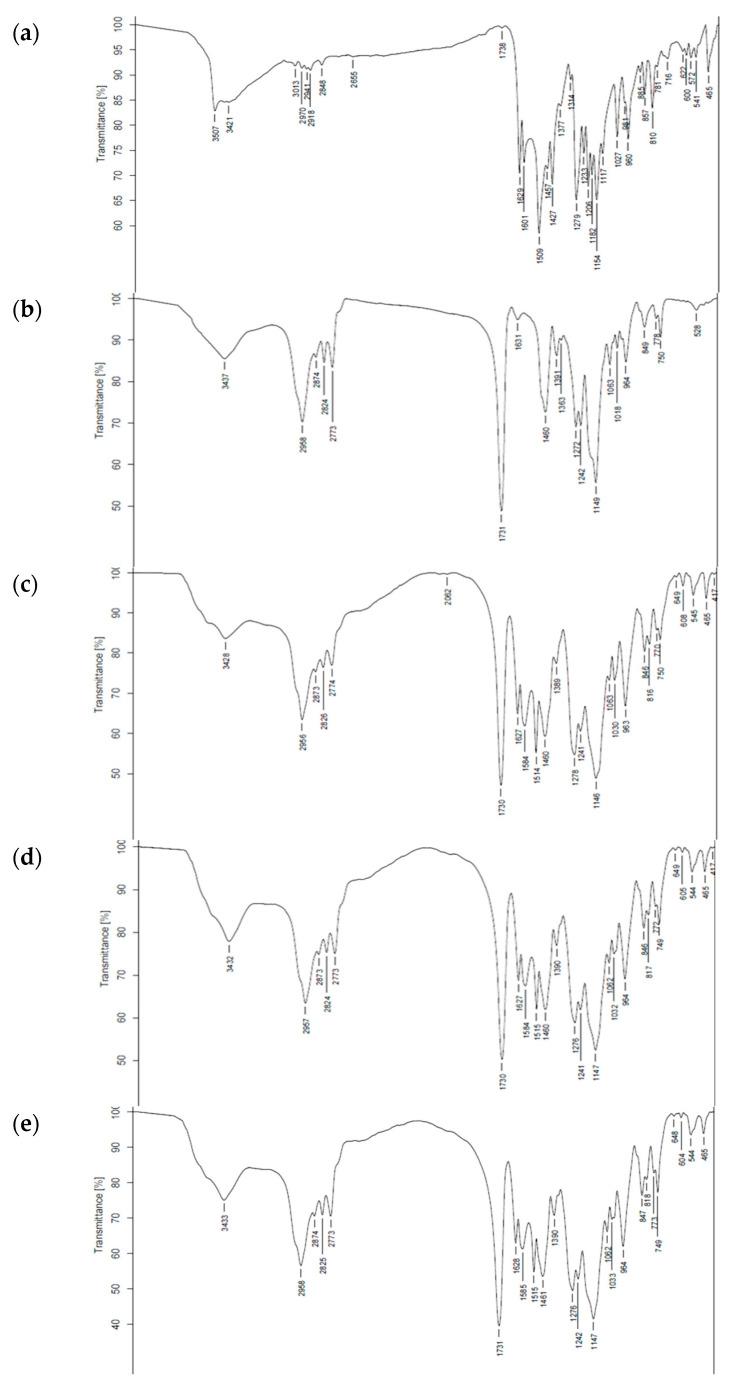

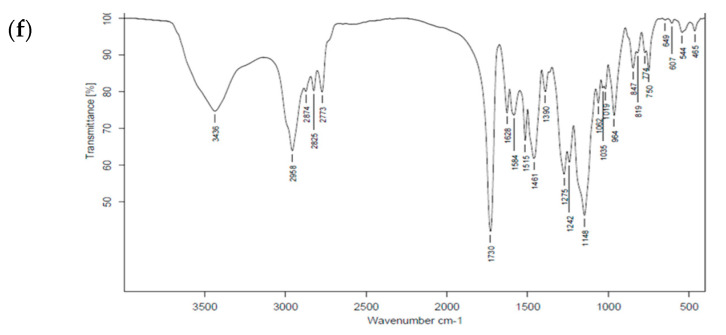
Fourier transform infrared spectra of (**a**) curcumin extract, (**b**) Eudragit EPO, and curcumin solid dispersion (CUR-SD) at *w/w* ratios of (**c**) 1:3, (**d**) 1:5, (**e**) 1:6, and (**f**) 1:8.

**Figure 3:**
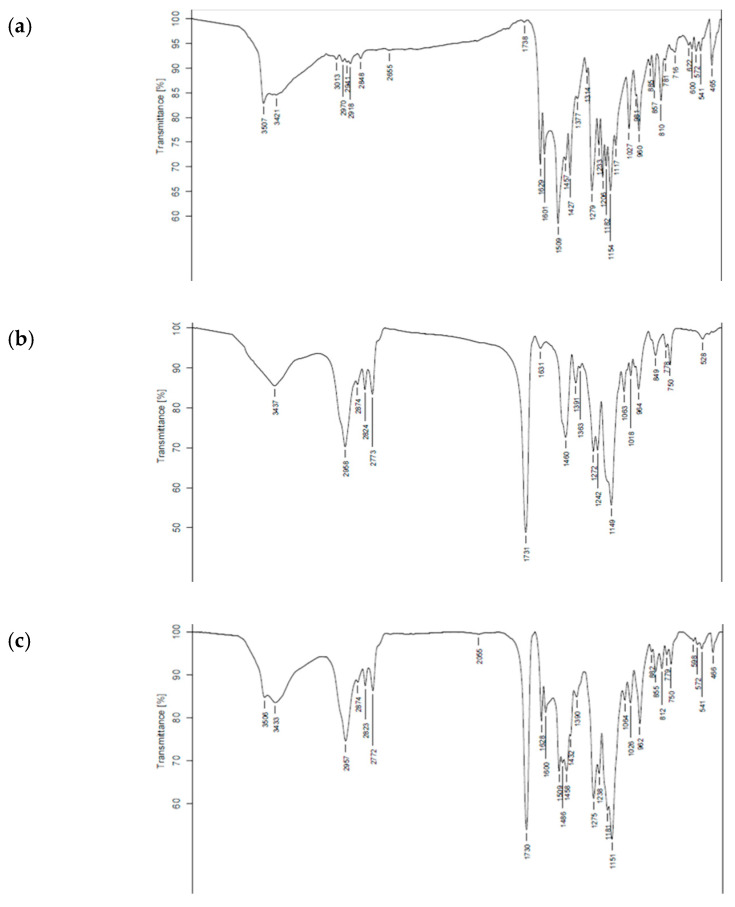

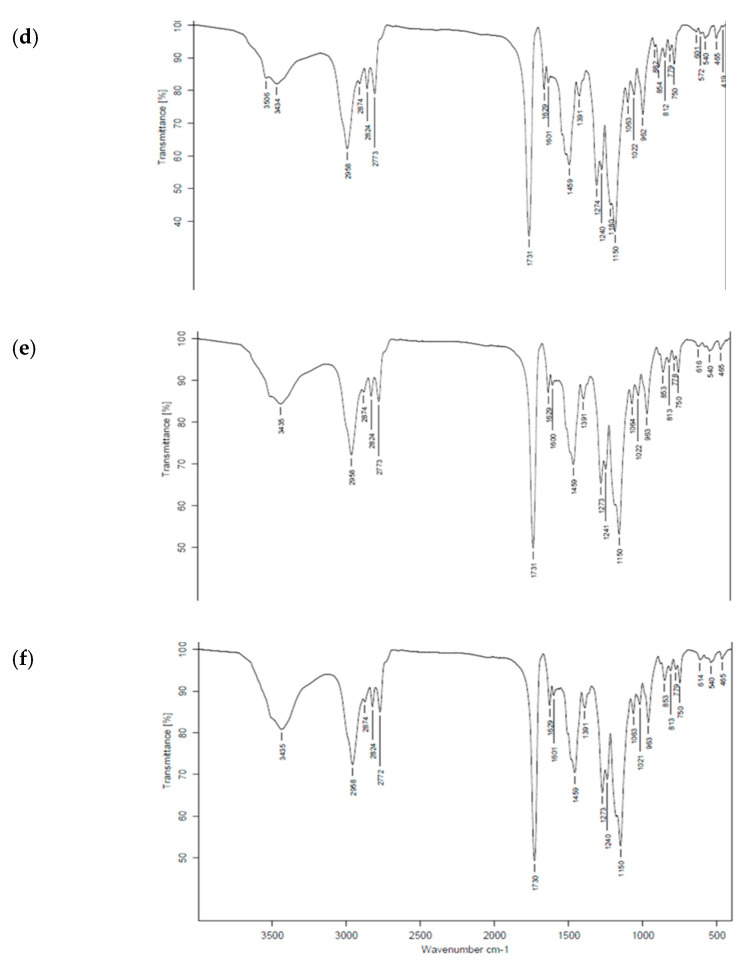
Fourier transform infrared spectra of (**a**) curcumin extract, (**b**) Eudragit EPO, and curcumin physical mixture (CUR-PM) at *w/w* ratios of (**c**) 1:3, (**d**) 1:5, (**e**) 1:6, and (**f**) 1:8.

FTIR spectroscopy was used to identify the chemical functional groups and interactions between curcumin and Eudragit polymer. As illustrated in Figure 2 and Figure 3, the FTIR spectrum of curcumin (Figure 2) presented a broad band at 3505–3447 cm^−1^ corresponding to an O-H stretching vibration in the polyphenol compound in the structure of curcumin. Eudragit^®^ EPO showed C=O stretching (carbonyl ester group) at 1731 cm^−1^, strong C-O absorption at 1149 cm^−1^, and a broad band with medium absorption due to amine (N-H) at 3437 cm^−1^ [35].

The FTIR spectra of all Cur EPO-PM (Figure 3) revealed a combination of peaks characteristic of curcumin and Eudragit, indicating an absence of interaction between these substances. In contrast, the FTIR spectrum of Cur EPO-SD (Figure 2) showed disappearance of the curcumin–O-H stretching vibration at 3505 cm^−1^, the N-H peak (3428–3436 cm^−1^), and the C=O stretching band (1730 cm^−1^) of Eudragit^®^ EPO, indicating strong intermolecular hydrogen bonding between hydroxyl groups in curcumin and cationic groups of Eudragit^®^ EPO. A similar interaction was reported previously for Cur EPO-SD [9] and Cur PVP-SD [36,37] and is considered to account for the transformation of curcumin from the crystalline to amorphous state, resulting in solubility enhancement.

### 2.4. X-Ray Powder Diffraction

**Figure 4:**
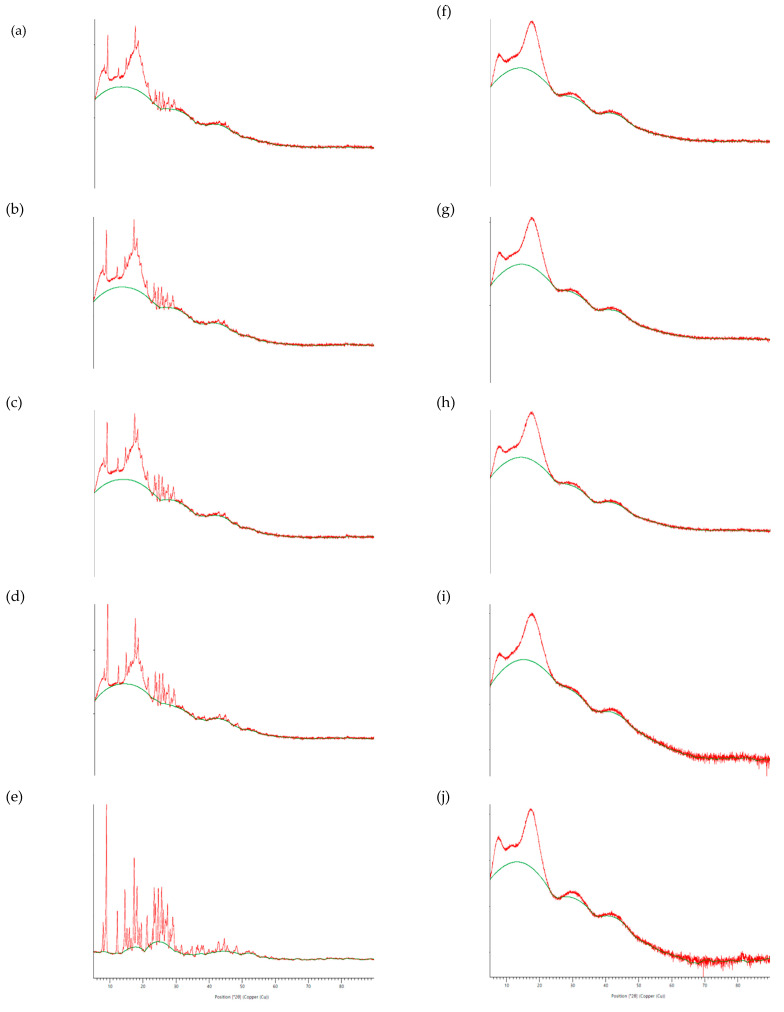
Powder X-ray diffractograms of curcumin physical mixture (CUR-PM) (**a**–**d**) in *w/w* ratios of (**a**) 1:8, (**b**) 1:6, (**c**) 1:5, and (**d**) 1:3; (**e**) curcumin extract; curcumin solid dispersion (CUR-SD) (**f**–**i**) in *w/w* ratios of (**f**) 1:8, (**g**) 1:6, (**h**) 1:5, and (**i**) 1:3; and (**j**) Eudragit EPO.

The powder X-ray diffractograms of curcumin, Eudragit^®^ EPO, Cur EPO-SD, and Cur EPO-PM are shown in Figure 4. Dominant peaks at diffraction angles (2θ) of 8.93°, 12.24°, 14.54°, 17.31°, 18.18°, 23.35°, 23.78°, 24.63°, and 25.62° confirmed the crystalline form of curcumin. On the other hand, the halo pattern of Eudragit^®^ EPO indicated the amorphous state of the polymer. The diffractograms of Cur EPO-PM displayed characteristic crystalline peaks of curcumin. In sharp contrast, crystalline peaks of curcumin were absent for all solid dispersion formulations, indicating the transformation of curcumin to the amorphous state. Eudragit^®^ EPO is considered to obstruct crystallization of curcumin by impeding the molecular motion through the polymer chain network and by hydrogen bonding interactions [9]. Similar findings of crystallinity inhibition have been reported for solid dispersions of curcumin-PVP K30 [36], curcumin–Solutol^®^HS [38,39], and glycoside-rich Centella asiatica extract–Eudragit^®^ EPO [10].

### 2.5. Physicochemical Characteristics of Curcumin-loaded Expandable Films

Corn starch, mung bean starch, pregelatinized starch, and banana starch were each blended with chitosan to prepare expandable film formulations containing Cur EPO-SD. The films were of uniform thickness, easily peeled from the casting substrate, and flexible to facilitate folding and insertion into hard gelatin capsules. The curcumin content of the expandable films was analyzed by UV spectroscopy. The average content was higher than 80% and ranged from 81 to 97%, demonstrating the high loading efficiency of the film formulations. Film weight varied depending on the amount of chitosan and starch used in the formulation (Table 2)—banana starch-based: 300–417 mg; corn starch: 315–437 mg; mung bean starch: 345–436 mg; and pregelatinized starch: 311–427 mg. Film thickness also correlated with the amount of chitosan and starch and ranged from 0.4 to 0.8 mm.

### 2.6. Determination of Mechanical Properties

The mechanical properties of the curcumin-loaded expandable films are important to avoid a breakdown in the stomach and to prolong residence time. Tensile strength is a crucial mechanical parameter that shows flexibility and strength of the films. However, in the field of gastroretentive drug delivery systems, no actual acceptable limits of tensile strength of film have been specified. Curcumin-loaded films prepared from mung bean starch displayed significantly higher tensile strength (90 kg/cm^2^) than those based on corn starch (35 kg/cm^2^), pregelatinized starch (42 kg/cm^2^), and banana starch (24 kg/cm^2^) (Table 2). Films with high tensile strengths are expected to have better resistance to breaking or disintegration for a substantial period during the drug release in the stomach than those with low tensile strength. Film tensile strength was highly influenced by chitosan content and decreased significantly when the weight of chitosan was reduced to below 600 mg. In comparison, changes in starch content exerted less effect on the tensile strength, as shown by the data obtained for films produced with a constant chitosan weight of 400 mg.

### 2.7. Film Swelling Studies

**Figure 5:**
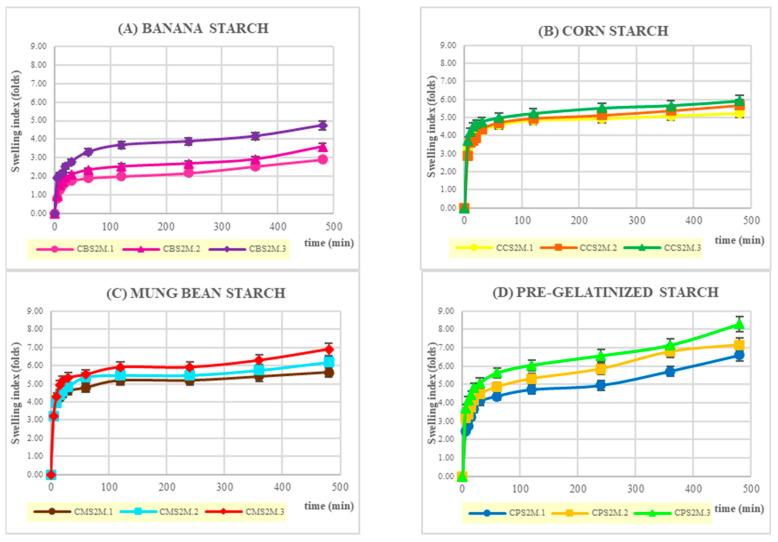
Swelling behavior of curcumin-loaded expandable films: (**A**) banana starch, (**B**) corn starch, (**C**) mung bean starch, and (**D**) pregelatinized starch.

The swelling behavior of curcumin-loaded expandable films in SGF (simulated gastric fluid) is shown in Figure 5. With the same amount of starch, the increment of chitosan tended to decrease the swelling index of banana starch, pregelatinized starch, and mung bean starch. However, in the case of corn starch, there was no difference in the swelling ratio at different levels of chitosan content. Pregelatinized starch showed the highest values of swelling ratio, whereas banana starch had lowest swelling index.

### 2.8. Examination of Film Morphology Using Scanning Electron Microscopy (SEM)

**Figure 6:**
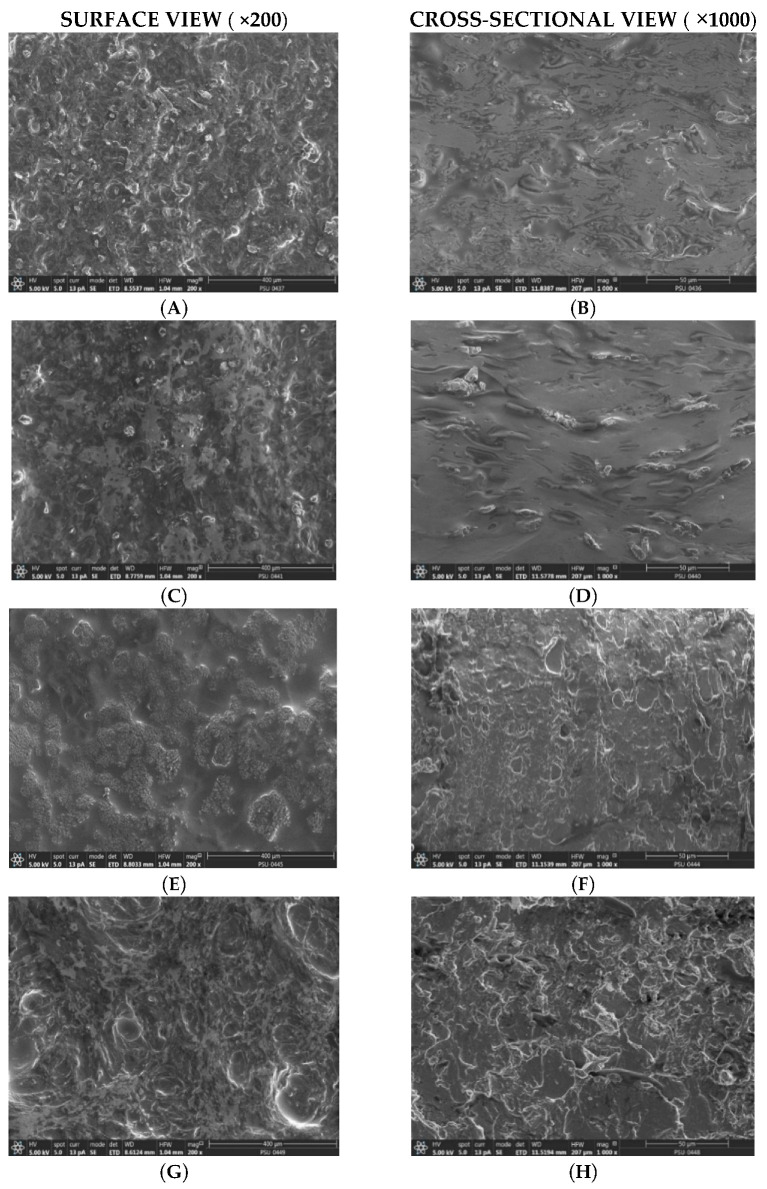
Scanning electron micrographs of curcumin-loaded expandable films: (**A**,**B**) banana starch (CBS2M.3), (**C**,**D**) corn starch (CCS2M.3), (**E**,**F**) mung bean starch (CMS2M.3), and (**G**,**H**) pregelatinized starch (CPS2M.3).

The morphologies of curcumin-loaded expandable films based on banana starch (Figure 6A,B) and corn starch (Figure 6C,D) were similar. The surface was rough and non-porous, with evidence of uniformly distributed curcumin solid dispersion. The film cross section showed a fibrous and porous structure, together with some embedded starch grains. The surface of films prepared using mung bean starch (Figure 6E) was non-porous, featuring numerous shallow pits or depressions.

The film cross section showed circular voids with no curcumin crystals in the film. Expandable films prepared from pregelatinized starch were characterized by a rough, porous surface texture (Figure 6G), whereas the cross section displayed smoother features with several voids.

### 2.9. Unfolding Behavior of Curcumin-Loaded Films

**Figure 7:**
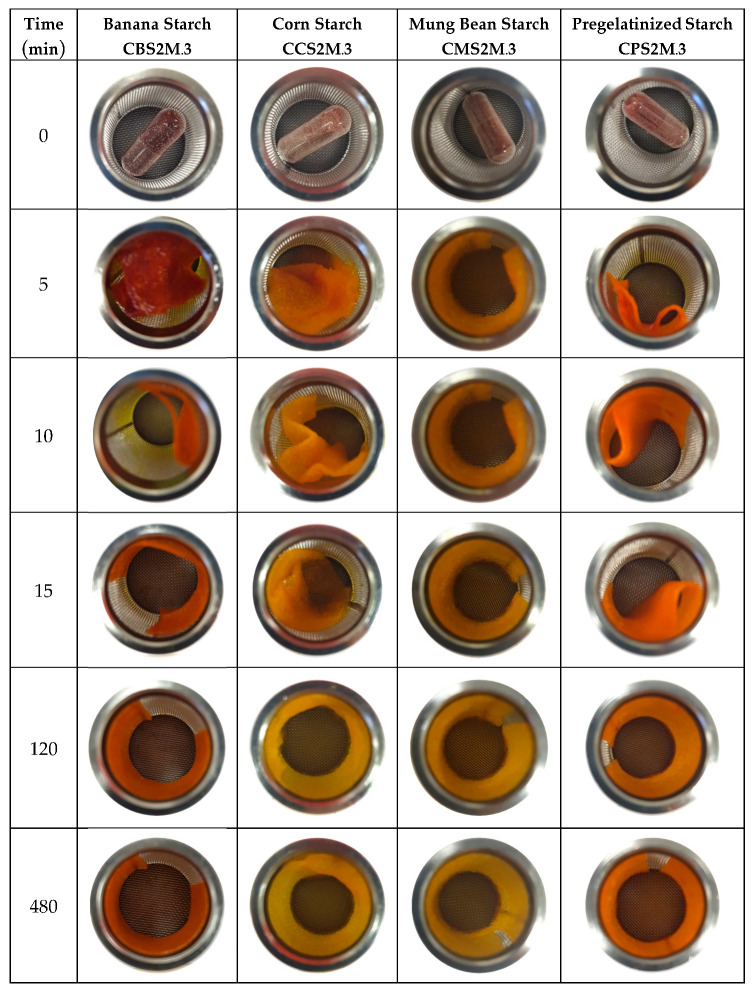
Unfolding and expansion behavior of curcumin-loaded films in simulated gastric fluid.

Curcumin-loaded films were folded in a zigzag pattern and inserted in hard gelatin capsules prior to exposure to SGF (simulated gastric fluid). Following dissolution of the capsules, the films gradually unfolded, and the process was complete within 1 h (Figure 7). The average time for the start of unfolding occurred in the following order: banana starch < corn starch < mung bean starch < pregelatinized starch. The film thickness gradually increased due to swelling, but films maintained a rectangular shape during 8 h of exposure to SGF. Film expansion (defined as the increase in film area) depended on film composition in the following order: corn starch (2.6–3.0 folds) > mung bean starch (2.5–3.3 folds) > pregelatinized starch (2.0–3.3 folds) > banana starch (1.5–2.0 folds), with a pyloric sphincter with a diameter of approximately 30 mm. All investigated formulations were found to be suitable gastroretentive dosage forms, owing to their rapid unfolding behavior and expansion characteristics in SGF.

### 2.10. In Vitro Curcumin Release from Expandable Films

**Figure 8:**
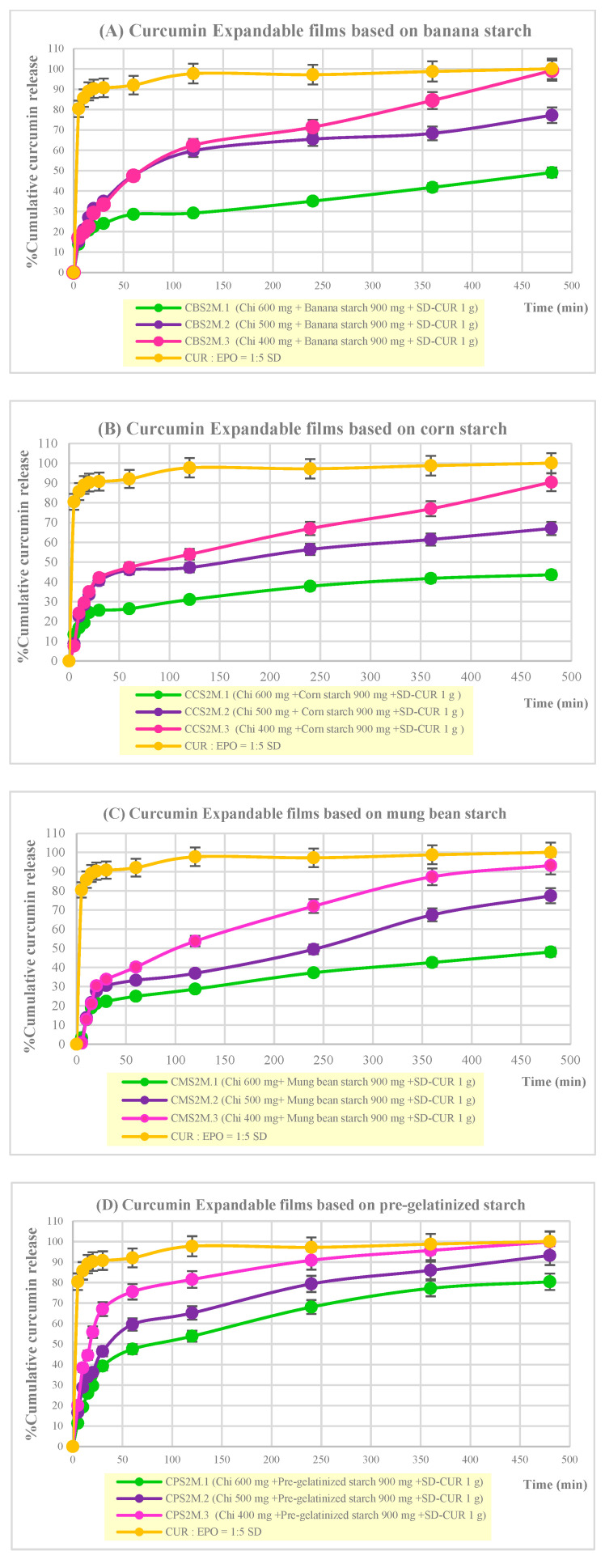
In vitro release of curcumin from expandable starch/chitosan films: (**A**) banana starch, (**B**) corn starch, (**C**) mung bean starch, and (**D**) pregelatinized starch.

Curcumin release from expandable starch/chitosan films generally exhibited a rapid burst release in the first hour, followed by a phase of gradual, sustained release up to 8 h (Figure 8). Formulations based on mung bean or corn starch resulted in the release of 20 –40% of the curcumin load in the first hour, whereas banana-starch-based films lost approximately 20–60% of the curcumin load over the same timescale. Decreasing the weight of chitosan in the films resulted in increasing cumulative release of curcumin. All curcumin-loaded film formulations, which contained 400 mg of chitosan and 900 mg of starch, resulted in highly efficient delivery of more than 80%. In comparison, film formulations based on 600 mg of chitosan resulted in low delivery efficiencies of less than 50%, except pre-gelatinized starch.

Films based on banana starch, corn starch, or mung bean starch resulted in 20–50% curcumin release in the first hour, followed by sustained release up to 8 h. On the other hand, expandable films based on pregelatinized starch exhibited high burst release in the first hour of 50–80%, which is a disadvantage for gastroretentive, sustained-release applications.

The kinetics of curcumin release from expandable films were analyzed using linear regression and by applying zero-order, first-order, Higuchi, Hixson–Crowell, and Korsmeyer–Peppas models. The correlation coefficients (r^2^) and release exponents (*n*) of selected formulations are listed in Table 3.

Curcumin release from expandable films based on banana starch (CBS2M.3), corn starch (CCS2M.3), and mung bean starch (CMS2M.3) closely followed the Higuchi release model (r^2^ = 0.9785, 0.9313, and 0.9684, respectively), indicating the operation of a diffusion-controlled process in a porous matrix system. Curcumin release may be envisaged through the fluid medium of the swollen starch/chitosan film, possibly facilitated by a porous film structure.

Curcumin release from films based on pregelatinized starch (CPS2M.3) closely followed the Korsmeyer–Peppas models, with a release exponent (r^2^) of 0.8434. The release exponent (n) was less than 0.45, indicating that curcumin release was controlled by Fickian diffusion through a polymeric matrix.

### 2.11. Cytotoxicity and Anti-inflammatory Activities of Curcumin Released from Expandable Films

Curcumin has been reported to exhibit anticancer activity against gastric cells by inhibiting cell proliferation and suppressing cell signaling pathways including COX-2 and NF-κB 40. The results of in vitro cytotoxicity testing using the MTT assay are shown in Table 4.

The IC_50_ value of Cur EPO-SD (ratio: 1:5) was 9.0 µg/mL, which was lower than that of unformulated curcumin (15.9 µg/mL), suggesting that the enhanced solubility of curcumin improves cellular uptake and therefore cytotoxicity activity. The cytotoxicity of curcumin incorporated in expandable starch/chitosan films was comparable to that of curcumin solid dispersion. IC_50_ values of curcumin-loaded film based on banana starch, corn starch, and mung bean starch were similar, namely 7.8 µg/mL, 8.1 µg/mL, and 7.3 µg/mL, respectively. These finding suggest that curcumin-loaded expandable films based on chitosan and starch show potential as gastroretentive dosage forms for the treatment of gastric cancer.

The anti-inflammatory activity of curcumin-loaded expandable films was evaluated in murine RAW 264.7 macrophages. Curcumin inhibits lipopolysaccharide-induced NO expression of inducible nitric oxide synthase (iNOS) and cyclooxygenase-2 (COX-2) [40]. Curcumin has also been shown to inhibit the release of proinflammatory cytokines, including tumor necrosis factor-α (TNF-α), interleukin-6 (IL-6), and activated nuclear factor kappa B (NF-κB).

Anti-inflammatory activity assay results showed IC_50_ values of 3.8 µg/mL and 3.0 µg/mL for curcumin and Cur EPO-SD, respectively, whereas indomethacin (positive control) exhibited a significantly higher IC_50_ of 20.3 µg/mL. The IC_50_ values of NO inhibitory activity of curcumin incorporated in expandable films based on banana starch (2.0 µg/mL), corn starch (2.1 µg/mL), banana starch (2.0 µg/mL), and mung bean starch (2.9 µg/mL) were comparable with those of curcumin solid dispersions.

These findings indicate the potential of curcumin-loaded films as gastroretentive formulations for the treatment of local conditions or to improve absorption and bioavailability.

## 3. Materials and Methods

### 3.1. Materials

Curcuminoid extract (96.1% curcumin) was obtained from Thai-China Flavours and Fragrances Industry Co., Ltd. (Phra Nakhon Si Ayutthaya, Thailand). Eudragit^®^ EPO was provided by Evonik Industries AG (Essen, Germany). Chitosan (molecular weight = 500,000; degree of deacetylation = 85%) was provided by Seafresh Industry Co., Ltd. (Bangkok, Thailand). Pregelatinized starch (Starch 1500^®^) was sourced from Colorcon, Inc. (Indianapolis, IN, United States).

Corn starch was purchased from Unilever Thai Holdings (Chachoengsao, Thailand), mung bean starch was purchased from Sitthinan Co., Ltd. (Pathumthani, Thailand), and banana flour (Musa sapientum Linn) was purchased from Thungpho PredPol Limited partnership (Phichit, Thailand). Glycerine and sodium chloride were provided by P.C. Drug Center, Co., Ltd. (Bangkok, Thailand). Glacial acetic acid was obtained from RCI Labscan (Bangkok, Thailand), and hard gelatin capsules (size 00) were sourced from Goldstar Chemical (Bangkok, Thailand). All other reagents used in this study were of analytical grade or pharmaceutical grade.

AGS cells (human gastric adenocarcinoma cell line) and RAW264.7 cells (murine leukemic macrophage cell line) were purchased from the American Type Culture Collection (ATCC; Manassas, Virginia, USA). DMEM (high-glucose Dulbecco’s Modified Eagle Medium) was obtained from Gibthai Co., Ltd. (Bangkok, Thailand). Fetal bovine serum (FBS; 10%) and trypsin EDTA (0.25%) were supplied by Gibco (Invitrogen, CA, USA). Penicillin and streptomycin were purchased from Invitrogen (Invitrogen, CA, USA). MTT reagent, phosphate-buffered saline (PBS; pH = 7.4), RPMI-1640 medium, lipopolysaccharide (LPS, from Escherichia coli), indomethacin, and Griess reagent were sourced from Sigma Aldrich (Sigma Aldrich, St. Louis, MI, USA). Dimethylsulfoxide (DMSO) was obtained from Amresco^®^ (Solon, OH, USA). All other chemicals were of analytical or pharmaceutical grade.

### 3.2. Preparation of Curcumin Solid Dispersions

Curcumin solid dispersions (CUR-SD) were prepared by the solvent evaporation method [9,41]. From curcumin and hydrophilic polymer (Eudragit^®^ EPO) at w/w ratios of 1:3, 1:5, 1:6, and 1:8. Briefly, curcumin (1 g) was dissolved in ethanol (500 mL), and the required weight of Eudragit^®^ EPO was added with stirring until completely dissolved. The solvent was removed by rotary evaporation (Hei-VAP Core, Heidolph Instruments GmbH, Schwabach, Germany) at 40 °C, and the resulting solid dispersion was dried under reduced pressure at 40 °C for 4–8 h. The dried samples of CUR-SD were pulverized using a glass mortar and pestle and sieved to obtain a 50–250 µm particle size fraction. Physical mixtures (CUR-PM) of curcumin and hydrophilic polymers were prepared at the same weight ratio as CUR-SDs by grinding in a mortar and pestle, followed by sieving to obtain a 50–250 µm particle size fraction. The CUR-SD and CUR-PM preparations were stored at room temperature in air-tight containers and protected from light until further use.

### 3.3. Investigation of the Physicochemical Properties of Curcumin Solid Dispersions

#### 3.3.1. Solubility Test

The solubility of curcumin when formulated as a solid dispersion was determined by the flask-shaking method [9] to determine the effect of the curcumin:polymer *w/w* ratio. Briefly, an excess amount of the sample (unformulated curcumin, CUR-SD, and CUR-PM) was added to a vial containing 1 mL 0.1 N hydrochloric acid (pH = 1.2) and agitated by vortex maxing (Vortex-Genie 2, 50 Hz model, Scientific Industries Inc., Bohemia, NY, USA) at maximum speed for 10 min. The mixtures were then shaken at 37 ± 0.1°C in a water bath shaker (SW22 Julabo, Seelbach, Germany) at a speed of 100 rpm for 48 h. Samples were centrifuged at 6000 rpm for 10 min.

The supernatants were withdrawn and filtered through a 0.45 μm pore size membrane filter, and the concentration of curcumin was analyzed by a UV-Visible spectrophotometer (UV-1800, Bara Scientific Co., Ltd., Bangkok, Thailand) at a wavelength of 425 nm. Samples were tested in triplicate (*n* = 3).

#### 3.3.2. In Vitro Dissolution Behavior

Curcumin dissolution behavior was investigated following the USP 41/ NF36 monograph using a Model EDT-08Lx rotating-paddle dissolution tester (Electrolab, Mumbai, India) at 37 ± 0.5 ℃ with a paddle rotation speed 50 rpm. Hydrochloric acid (volume: 900 mL; 0.1 N; pH: 1.2; simulated gastric fluid (SGF) was used as the dissolution medium. Samples (100 mg) of unformulated curcumin, CUR-SD, or CUR-PM were added to the dissolution, and 5 mL aliquots were withdrawn and replaced with fresh medium after 5, 10, 30, 45, 60, 90, and 120 min. The amount of curcumin in the withdrawn samples was measured by a UV spectrophotometer at a wavelength of 425 nm. Each formulation was tested in triplicate (*n* = 3), and data were reported as a mean ± S.D. A plot of cumulative release (% *w/w*) of curcumin against time (min) was constructed to illustrate the curcumin dissolution profiles.

#### 3.3.3. Fourier Transform Infrared Spectroscopy Studies

Fourier transform infrared spectroscopy (FTIR) was performed to identify the chemical interaction between curcumin and the hydrophilic polymers used for the preparation of CUR-SDs and CUR-PMs [9]. Samples were compressed into potassium bromide (KBr) disks and scanned using an FTIR spectrometer (VERTEX 70, Bruker, Ettlingen, Germany) equipped with a deuterated triglycine sulfate detector. Spectra were obtained over the range of 400 to 4000 cm^−1^ at a resolution of 4 cm^−1^.

#### 3.3.4. X-Ray Powder Diffraction

The crystallinity of unformulated curcumin, CUR-SD, CUR-PM, and the hydrophilic polymers (Eudragit^®^ EPO) was analyzed by X-ray powder diffraction (Philips: X’ pert MPD, Empyrean, PANalytical, Netherlands) at room temperature with a scan speed of 1 s/step over a 2θ range of 5–90° and a step size of 0.05° for 2 h.

### 3.4. Preparation of Starch/Chitosan Expandable Films Loaded with Curcumin Solid Dispersion

Expandable films loaded with curcumin solid dispersion were prepared by solvent casting method using blends of chitosan and mung bean starch, pregelatinized starch, corn starch, and banana starch. The content of chitosan was varied systematically from 400 mg to 600 mg (Table 2) and mixed with 600–1000 mg of each type of starch. Glycerin was used as a plasticizer.

In brief, starch and chitosan were weighed in the required amounts and dissolved in 35 mL of 2% *v/v* acetic acid solution. The mixture was heated to 100 °C with continuous stirring for 60 min to gelatinize the starch component and cooled to room temperature. Glycerin (900 mg) and 1 g of curcumin–Eudragit^®^ EPO solid dispersion (ratio 1:5) were added and mixed for 4 h to produce a homogeneous solution. Films were cast by pouring the solution into a glass Petri dish and dried at 40 °C for 48 h. The obtained films were carefully removed from the Petri dish and stored in light-resistant containers at room temperature. Test samples were dusted with microcrystalline cellulose (MCC) as an antiadhesive prior to testing.

### 3.5. Evaluation of the Physicochemical Properties of Curcumin-Loaded Expandable Films

#### 3.5.1. Film Weight, Thickness, and Curcumin Loading

Samples of curcumin-loaded films (40mm × 20 mm) were dissolved in 100 mL of methanol and sonicated for 30 min in an ultrasonic bath to extract curcumin. Aliquots were filtered using filter paper (Whatman™ no. 1; diameter: 125 mm) and diluted to a suitable concentration before measurement of the curcumin concentration by UV spectrophotometry at a 425 nm wavelength. The variation of film weight was obtained by measuring samples (40 mm × 20 mm) using a digital analytical balance (PRACTUM224-1S, Sartorius Lab Instruments GmbH&Co., Ltd., Goettingen, Germany). Each formulation was tested in triplicate, and results were reported as mean ± S.D. Film thickness was measured using a vernier caliper (V6-154, Kovet Co, Ltd., Bangkok, Thailand) with an accuracy of ±0.001 mm. Values were reported as mean ± S.D.

#### 3.5.2. Film Tensile Strength

Film tensile strength (TS) was measured using a texture analyzer (TA.XT plus, Stable Micro Systems; Charpa Techcenter Co. Ltd., Bangkok, Thailand) equipped with a 25 kg load cell following the method described by Kaewkroek et al. [12,42]. Rectangular test samples (60 × 10 mm) free of air bubbles and other physical imperfections were clamped 30 mm apart and extended at a rate of 2.0 mm/s until the film ruptured. The maximum applied load was recorded, and the tensile strength was calculated using the following equation. Each type of film was tested in triplicate, and the tensile strength was expressed as mean ± S.D.

Tensile strength (kg/cm^2^) = force at break (kg)/initial cross-sectional area of the sample (cm^2^)

#### 3.5.3. Film Swelling Behavior

Triplicate samples of curcumin-loaded expandable films (10 mm × 20 mm) were weighed and immersed in 0.1 N hydrochloride acid solution (pH = 1.2) at 37 ± 0.5 °C. After 8 h, the film was removed from the medium and weighed again. The swelling ratio was calculated using the following equation:

% Swelling ratio = [ (W_1_−W_0_)/W_0_] × 100

where W_0_ is the weight of the dry sample, and W_1_ is the weight of the sample after immersion in the medium. Swelling ratios were reported as mean ± S.D.

#### 3.5.4. Examination of Film Morphology Using Scanning Electron Microscopy (SEM)

The morphologies of the surface and cross section of curcumin-loaded films were visualized using a field-emission scanning electron microscope (FEI-SEM Apreo, Eindhoven, the Netherlands). Each sample was coated with a thin layer of gold before analysis, and SEM images were obtained at an operating voltage of 20 kV.

#### 3.5.5. Fourier Transform Infrared (FTIR)

Fourier transform infrared spectroscopy was performed on samples of curcumin-loaded films to identify the chemical structure and chemical interactions. Spectra were recorded in the range of 4000 to 400 cm^−1^ at a resolution of 4 cm^−1^.

#### 3.5.6. Powder X-Ray Diffraction Studies of Curcumin-Loaded Expandable Films

The crystallinity of curcumin in expandable films was analyzed by X-ray diffraction as previously described for CUR-SDs and CUR-PMs.

#### 3.5.7. Unfolding Behavior of Curcumin-Loaded Films

Samples of expandable films (40 mm × 20 mm) were folded in a zigzag pattern before insertion into hard gelatin capsules (size 00). The unfolding behavior was investigated using a rotating basket dissolution apparatus. The films were removed from the baskets at predesignated time intervals, and their appearance and dimensions were recorded.

#### 3.5.8. In Vitro Release of Curcumin from Expandable Films

In vitro curcumin release profiles were investigated using a rotating basket dissolution apparatus (900 mL of 0.1 N hydrochloric acid; pH = 1.2) at 37 ± 0.5 ℃ with a rotation speed 50 rpm. Hard gelatin capsules containing curcumin-loaded films were added into the release medium, and samples (5 mL) were withdrawn and replaced with fresh medium after 5, 10, 15, 20, 30, 60, 120, 240, 480, and 720 min. The concentration of curcumin in the release medium was measured by a UV spectrophotometer at a wavelength of 425 nm. Each formulation was tested in triplicate and reported as mean ± S.D. Curcumin release profiles were constructed in the form of % cumulative release versus time. The release data were plotted according to the zero-order, first-order, and Higuchi models to establish the release kinetics and mechanism [43,44]. A coefficient of determination (r^2^) closer to 1 indicated the best fit of release data to a particular kinetic model.

### 3.6. In Vitro Cytotoxicity Assay of Curcumin-Loaded Expandable Films

The cytotoxic activity of curcumin-loaded films towards AGS cells (human gastric adenocarcinoma cell line; ATCC^®^ CRL¬1739™) was investigated by MTT assay. MTT (3- (4,5-dimethylthiazol-2-yl) -2,5-diphenyltetrazolium bromide) (yellow tetrazole) is modified by mitochondrial reductase enzymes to formazan (purple), which is quantifiable by colorimetric assay. Briefly, AGS cells were seeded at a density of 2 × 104 cells/well in 96-well plates in DMEM (Gibco^®^; 4.5 g/L glucose) supplemented with 10% fetal bovine serum (FBS), 100 µg/mL streptomycin, and 100 U/mL penicillin at 37°C in a humidified 5% carbon dioxide (CO_2_) atmosphere. After 24 h incubation, the medium culture was removed, and 100 μL of test samples of curcumin, curcumin solid dispersion, and curcumin-loaded expandable films containing a set concentration of curcumin were added to the cells. The control group consisted of cell culture medium with fetal bovine serum. After 24, 48, and 72 h of incubation, 20 µL of MTT solution (5 mg/mL in PBS) was added to each well, and incubation was continued for more than 4 h. Following removal of the supernatant, 200 μL of dimethyl sulfoxide (DMSO) was added to each well to solubilize the purple formazan crystals. Absorbance was measured at a wavelength of 570 nm with a microplate reader (Biotek model Power Wave X, Santa Clara, CA, USA). [29,45,46].

Antiproliferative activity was expressed in terms of percentage cell viability according to the following equation:

Cell Viability (%) = [ (OD_570nm_ of cell in each well)/(Mean OD_570nm_ of control cells) ] × 100

The IC_50_ value of each formulation was calculated as the concentration of curcumin that inhibited cell proliferation by 50%. All experiments were performed in triplicate.

### 3.7. Evaluation of Anti-Inflammatory Properties of Curcumin Expandable Films

The anti-inflammatory effect of curcumin-loaded films was assessed by measurement of nitric oxide (NO) production by RAW264.7 cells (murine leukemic macrophage cell lines). Cells were seeded at a density 1X10^5^ cells per well in 96–well plates in RPMI medium at 37 ℃ and a 5% CO_2_ environment. The culture medium contained 0.1% sodium bicarbonate, 2 mM glutamine, 100 units/mL penicillin G, 100 µg/mL streptomycin, and 10% FCS. Following incubation for 24 h, the medium was removed and replaced with a fresh medium containing 100 µg/mL lipopolysaccharides (LPS). Test samples comprising CUR-SD, curcumin-loaded expandable films, and unformulated curcumin containing set concentrations of curcumin (0–25 µg/mL) were added to the cells and incubated for 48 h. Indomethacin was tested as a positive control (0–100 µg/mL). The supernatant was collected and mixed with Griess reagent, and the absorbance at 570 nm was measured in order to detect NO production by accumulation of nitrite. Anti-inflammatory activity was expressed as percentage inhibition of NO as calculated using the following equation. Indomethacin was applied as a positive control.

Inhibition (%) = [((A−B))/((A−C))] × 100

where A–C is the NO_2_ concentration (µM); A is LPS (+), sample (−); B is LPS (+), sample (+); and C is LPS (−), sample (−).

### 3.8. Statistical Analysis

All data are reported as mean ± standard deviation. Data were subjected to one-way analysis of variance (one-way ANOVA) using the SPSS program, and statistical significance was accepted at *p*-values less than 0.05.

## 4. Conclusions

Gastroretentive expandable films incorporating curcumin–Eudragit solid dispersions were prepared based on starch/chitosan blends. Films unfolded completely within 1 h when inserted in hard gelatin capsules and exposed to simulated gastric fluid (SGF). Sustained release of curcumin occurred from the films in SGF over 8 h, resulting in highly efficient delivery in excess of 80%. Curcumin release from expandable films resulted in significantly greater cytotoxic activity against gastric cancer cells than free curcumin and higher anti-inflammatory activity against RAW 264.7 macrophage cells than indomethacin. These findings demonstrate the potential of expandable curcumin-loaded films as gastroretentive dosage forms for the treatment of gastric diseases and to improve oral bioavailability.

## Figures and Tables

**Table 1:** The solubility of curcumin from curcumin solid dispersions and their physical mixtures at various ratios (1:3, 1:5, 1:6, and 1:8).

Formulation and Ratio	Solubility of Curcumin (mg/mL)	
Curcumin	0.000527 ± 0.000002	
CUR: Eudragit EPO	Solid Dispersion	Physical Mixture
1:3	4.10 ± 0.04	0.44 ± 0.02
1:5	6.38 ± 0.10	1.20 ± 0.03
1:6	3.70 ± 0.05	0.79 ± 0.01
1:8	2.62 ± 0.03	0.66 ± 0.01

**Table 2:** Composition and properties of curcumin-loaded expandable films.

Formulations		Weight of Excipient				Tensile Strength (kg/cm^2^)	Expansion* (fold)	Drug Content (%)
		Chitosan (mg)	Starch (mg)	Film Weight (mg)	Film Thickness (mm)			
**Banana** **Starch**	CBS2M.1	600	900	416.90 ± 0.75	0.789 ± 0.014	24.48 ± 0.42	1.97 ± 0.02	84.85 ± 0.19%
	CBS2M.2	500	900	405.70 ± 1.40	0.539 ± 0.007	6.36 ± 0.07	2.01 ± 0.02	81.63 ± 0.05%
	CBS2M.3	400	900	383.87 ± 0.25	0.478 ± 0.007	9.07 ± 0.18	2.02 ± 0.02	85.31 ± 0.04%
	CBS2M.4	400	800	362.73 ± 1.24	0.514 ± 0.009	7.13 ± 0.03	1.97 ± 0.02	84.44 ± 0.17%
	CBS2M.5	400	700	335.40 ± 1.42	0.482 ± 0.006	6.82 ± 0.09	1.90 ± 0.02	88.64 ± 0.15%
	CBS2M.6	400	600	299.63 ± 0.82	0.453 ± 0.008	9.47 ± 0.12	1.53 ± 0.00	87.99 ± 0.04%
	CBS2M.7	400	1000	392.83 ± 0.45	0.588 ± 0.007	12.97 ± 0.18	1.54 ± 0.01	86.46 ± 0.05%
**Corn** **Starch**	CCS2M.1	600	900	437.00 ± 1.90	0.516 ± 0.007	35.34 ± 0.02	2.57 ± 0.02	97.04 ± 0.58%
	CCS2M.2	500	900	426.40 ± 1.71	0.492 ± 0.009	15.04 ± 0.29	2.62 ± 0.02	88.27 ± 0.19%
	CCS2M.3	400	900	414.67 ± 1.80	0.456 ± 0.005	7.59 ± 0.13	2.83 ± 0.02	85.00 ± 0.40%
	CCS2M.4	400	800	363.83 ± 0.29	0.535 ± 0.005	10.27 ± 0.07	3.00 ± 0.02	90.98 ± 0.33%
	CCS2M.5	400	700	351.13 ± 1.62	0.506 ± 0.005	9.50 ± 0.14	2.95 ± 0.02	88.30 ± 0.14%
	CCS2M.6	400	600	314.47 ± 1.47	0.480 ± 0.008	8.98 ± 0.49	2.88 ± 0.02	86.74 ± 0.11%
	CCS2M.7	400	1000	433.87 ± 0.29	0.556 ± 0.007	8.25 ± 0.12	2.97 ± 0.01	96.00 ± 0.07%
**Mung** **Bean** **Starch**	CMS2M.1	600	900	436.27 ± 1.14	0.505 ± 0.005	90.43 ± 14.04	2.47 ± 0.02	81.49 ± 0.01%
	CMS2M.2	500	900	426.20 ± 1.73	0.475 ± 0.005	28.77 ± 5.71	2.75 ± 0.02	88.46 ± 0.31%
	CMS2M.3	400	900	388.60 ± 1.07	0.415 ± 0.005	14.16 ± 0.59	2.53 ± 0.02	84.02 ± 0.25%
	CMS2M.4	400	800	383.30 ± 0.41	0.532 ± 0.009	13.90 ± 2.26	2.82 ± 0.02	81.34 ± 0.05%
	CMS2M.5	400	700	367.73 ± 1.86	0.482 ± 0.007	11.87 ± 1.05	3.32 ± 0.01	89.59 ± 0.13%
	CMS2M.6	400	600	345.00 ± 1.04	0.439 ± 0.008	9.78 ± 0.27	2.91 ± 0.02	83.32 ± 0.05%
	CMS2M.7	400	1000	433.60 ± 1.78	0.608 ± 0.012	14.12 ± 0.15	2.59 ± 0.01	87.69 ± 0.07%
**Pregelatinized** **starch**	CPS2M.1	600	900	427.07 ± 0.56	0.680 ± 0.009	42.40 ± 0.79	1.95 ± 0.02	96.97 ± 1.37%
	CPS2M.2	500	900	394.00 ± 0.49	0.580 ± 0.010	7.84 ± 0.01	2.30 ± 0.02	81.79 ± 0.52%
	CPS2M.3	400	900	387.30 ± 1.87	0.512 ± 0.004	8.45 ± 0.13	2.43 ± 0.02	81.72 ± 0.06%
	CPS2M.4	400	800	340.53 ± 1.59	0.586 ± 0.005	7.70 ± 0.19	2.81 ± 0.02	85.87 ± 0.05%
	CPS2M.5	400	700	328.13 ± 0.31	0.550 ± 0.008	8.72 ± 0.58	3.22 ± 0.01	85.23 ± 0.07%
	CPS2M.6	400	600	310.63 ± 1.73	0.479 ± 0.008	9.25 ± 0.69	3.31 ± 0.02	84.17 ± 0.13%
	CPS2M.7	400	1000	405.33 ± 1.10	0.606 ± 0.010	8.53 ± 0.31	2.76 ± 0.02	84.40 ± 0.03%

Films containing 900 mg glycerin as a plasticizer and 1000 mg curcumin solid dispersion. Expansion* (fold) = area of the film after test/area of the film before test.

**Table 3:** Linear regression of curcumin release from expandable films based on starch and chitosan.

Formulation	Drug Release Kinetic, Correlation Coefficient (r^2^)				Release Exponent (*n*)
	Zero Order	First Order	Higuchi	Korsmeyer–Peppas	
Banana starch (CBS2M.3)	0.8695	0.7657	0.9785	0.8982	0.3978
Corn starch (CCS2M.3)	0.8010	0.5266	0.9313	0.8338	0.4113
Mung bean starch (CMS2M.3)	0.8612	0.3483	0.9684	0.6713	0.7675
Pregelatinized starch (CPS2M.3)	0.5919	0.4128	0.7964	0.8434	0.2923

**Table 4:** In vitro anti-inflammatory and cytotoxicity activity of curcumin-loaded expandable films.

Test Sample	Anti-NO Test IC_50_ (µg/mL)	Cytotoxicity IC_50_ (µg/mL)
Banana-starch-based film (CBS2M.3)	2.0 ± 0.8 *	7.8 ± 1.3
Blank banana starch (BB)	>12.5	>100
Corn-starch-based film (CCS2M.3)	2.1 ± 0.9 *	8.1 ± 1.9
Blank corn starch (CB)	>12.5	>100
Mung bean starch-based film (CMS2M.3)	2.9 ± 0.8 *	7.3 ± 2.1
Blank mung bean starch (MB)	>12.5	>100
SD-CUR	3.0 ± 1.5	9.0 ± 0.9
Curcumin	3.8 ± 1.1	15.9 ± 1.6
Indomethacin	20.3 ± 1.8	-

* Each value represents mean ± S.D. of four determinations.

## Data Availability

Data and results are reported in this article.

## Sample Availability

Samples of the compounds are not available from the authors.
